# Perspectives on the Nutritional Paradox in Uremia: A “Sensitize-Then-Feed” Framework for Protein-Energy Wasting^☆^

**DOI:** 10.1016/j.advnut.2026.100728

**Published:** 2026-09-09

**Authors:** Maoting Li, Zewei Chen, Nanmei Liu, Naiying Lan, Fanzhou Zeng, Cheng Xue, Bo Yang

**Affiliations:** 1 Department of Nephrology, Naval Medical Center of PLA, Naval Medical University, Shanghai, China; 2 Department of Cellular Biology and Anatomy, Medical College of Georgia, Augusta University, Augusta, GA, United States; 3 Department of Nephrology, Shanghai Changzheng Hospital, Second Affiliated Hospital of Naval Medical University (SMMU), Shanghai, China

**Keywords:** hemodialysis, protein-energy wasting (PEW), ghrelin-GOAT axis, uremic toxins, nutritional therapy, anabolic resistance

## Abstract

Protein-energy wasting (PEW) drives significant morbidity and mortality in advanced chronic kidney disease (CKD). Despite the implementation of comprehensive clinical guidelines and medical nutrition therapy, a subset of uremic patients may exhibit a defining “nutritional paradox.” Unlike simple starvation, which responds to caloric and protein supplementation, CKD-associated cachexia is characterized by a potential systemic anabolic resistance. This renders standard nutritional interventions—including oral supplements and intradialytic parenteral nutrition—often insufficient. To elucidate the molecular hierarchy driving this barrier to nutritional therapy, we propose a model in which, during central dysregulation, gut-microbiome–derived uremic toxins, notably tryptophan metabolites, such as indoxyl sulfate, have been shown in experimental models to impair the blood–brain barrier integrity. This breach triggers significant hypothalamic neuroinflammation. Concurrently, proinflammatory cytokines (e.g., interleukin-1*β*, tumor necrosis factor–*α*) and stress-responsive circulating factors like growth differentiation factor 15 (GDF15) aberrantly reset the central metabolic set point via the melanocortin system, potentially contributing to anorexia and altered energy expenditure. Peripherally, the failure of critical nutrient-sensing axes—specifically, the ghrelin–ghrelin O-acyltransferase and insulin/insulin-like growth factor–1 signaling pathways—may impair the anabolic phosphoinositide 3-kinase/Akt/mechanistic target of rapamycin complex 1 cascade. This peripheral desensitization not only blunts the synthetic response to dietary amino acids but also precipitates a disruption of muscle proteostasis through ubiquitin–proteasome system hyperactivation and forkhead box O–mediated transcription. Concurrently, skeletal muscle undergoes significant mitochondrial bioenergetic dysfunction, further impairing nutrient utilization. By reframing PEW not simply as a state of nutrient deficiency, but as a broader dysregulation of hormonal signaling, we advocate a translational “sensitize-then-feed” therapeutic paradigm. We critically evaluate emerging targeted agents—including GDF15 antagonists, myostatin inhibitors, and ghrelin agonists. We propose that these agents could be investigated as metabolic modulators to potentially enhance the efficacy of tailored nutritional support in the uremic microenvironment.

## Introduction: The Nutritional Paradox in Chronic Kidney Disease

**Statement of significance**

Although nutritional supplementation remains an important component of the management of protein-energy wasting in chronic kidney disease, a subset of patients may exhibit limited anabolic and clinical responses to feeding alone. By integrating emerging evidence from human studies and experimental models, this perspective article proposes a testable “sensitize-then-feed” neuroendocrine framework to understand these metabolic barriers and guide future translational and clinical research.

As chronic kidney disease (CKD) progresses to end-stage renal disease (ESRD), progressive wasting of body protein and energy stores may persist despite standard nutritional interventions [1]. Protein-energy wasting (PEW) [2] represents an uncoupling of nutrient intake from anabolic responses. Despite the widespread availability of renal replacement therapies and sophisticated dietary formulations, PEW remains a potent predictor of morbidity and mortality [3], affecting between 11% and 54% of the advanced CKD population [4].

The classical understanding of CKD wasting was centered on anorexia driven by uremic toxins and restrictive renal diets. The logical therapeutic corollary was increasing nutrient provision through oral nutritional supplements (ONS), intradialytic parenteral nutrition (IDPN), or enteral feeding [5,6]. However, decades of clinical experience and randomized trials have demonstrated that simply providing adequate or even supraphysiological calories and protein often fails to arrest the catabolic trajectory or restore lean muscle mass in patients with uremia [7]. Unlike simple starvation, where metabolic machinery adapts to conserve protein [8], the uremic phenotype is a maladaptive hypercatabolic state [9]. The body metabolizes its own skeletal muscle despite exogenous nutrients.

This perspective article proposes that the core lesion in CKD-associated wasting may extend beyond substrate deficiency to involve dysregulation of hormonal signaling. This resistance appears to manifest as a blockade at multiple checkpoints in the neuroendocrine axis: centrally, where the brain becomes desensitized to hunger signals and hypersensitive to inflammatory stress; and peripherally, where skeletal muscle becomes refractory to anabolic hormones, such as insulin and insulin-like growth factor–1 (IGF-1), while remaining acutely sensitive to catabolic drivers [10,11]. However, it is important to note that “hormonal resistance” is used here as a broad conceptual umbrella. Biologically, this encompasses 2 distinct pathophysiological processes: true anabolic resistance, characterized by impaired receptor or postreceptor signaling [e.g., insulin/IGF-1 resistance via insulin receptor substrate 1 (IRS-1) phosphorylation], and pathological hyperactivation, where catabolic or anorexigenic pathways [e.g., GDF15–Glial cell line-derived neurotrophic factor (GDNF) family receptor *α*-like (GDF15-GFRAL), melanocortin-4 receptor (MC4-R)] are inappropriately upregulated despite energy deficits.

Emerging research has significantly clarified the molecular mechanisms underpinning this resistance. We now recognize a pathological sequence driven by the disruption of the blood–brain barrier (BBB) by uremic toxins, the dysregulation of the ghrelin–ghrelin O-acyltransferase (GOAT) enzyme system, and the accumulation of stress cytokines like GDF15 [[12], [13], [14]]. This resistance extends beyond protein turnover to include mitochondrial bioenergetic failure and defective mitophagy, creating a “metabolic deadlock” that nutritional therapy alone cannot break [15,16].

### Search strategy and selection criteria

Data for this perspective article were identified by comprehensive searches of PubMed, MEDLINE, and Embase, alongside cross-referencing from relevant articles. We considered literature published from database inception up to February 2026. The search strategy utilized combinations of terms related to the renal condition (“chronic kidney disease,” “end-stage kidney disease,” “uremia,” “uremic toxins,” and “indoxyl sulfate”) and the metabolic phenotype (“protein-energy wasting,” “cachexia,” “sarcopenia,” “anabolic resistance,” and “hormonal resistance”). To capture the specific mechanistic and therapeutic scopes of this perspective article, additional queries incorporated “ghrelin,” “GDF15,” “mitochondrial dysfunction,” “ubiquitin–proteasome system,” and the “gut–brain–muscle axis.”

We selected English-language literature based on predefined considerations, including relevance to the central mechanistic questions addressed in this perspective article, study design, and evidentiary strength. This included recent mechanistic studies, randomized controlled trials, robust observational cohorts, and recent meta-analyses that elucidate the neuroendocrine-metabolic pathways of uremic muscle wasting. Seminal historical papers and consensus statements were also included to provide essential conceptual background and trace the evolution of the “nutritional paradox” in nephrology. The final reference list was generated on the basis of originality and direct relevance to the proposed mechanistic framework.

## The Neuroendocrine Mechanism

The pathogenesis of PEW in CKD is driven by a synchronous failure in 2 critical regulatory systems: central regulation of appetite and peripheral regulation of nutrient utilization (Figure 1, Table 1). Central dysregulation (anorexia) may manifest earlier in the CKD continuum because of toxin accumulation [17], whereas peripheral anabolic resistance (muscle wasting) may become the dominant driver in ESRD as mitochondrial damage and insulin resistance compound over time.

**FIGURE 1:**
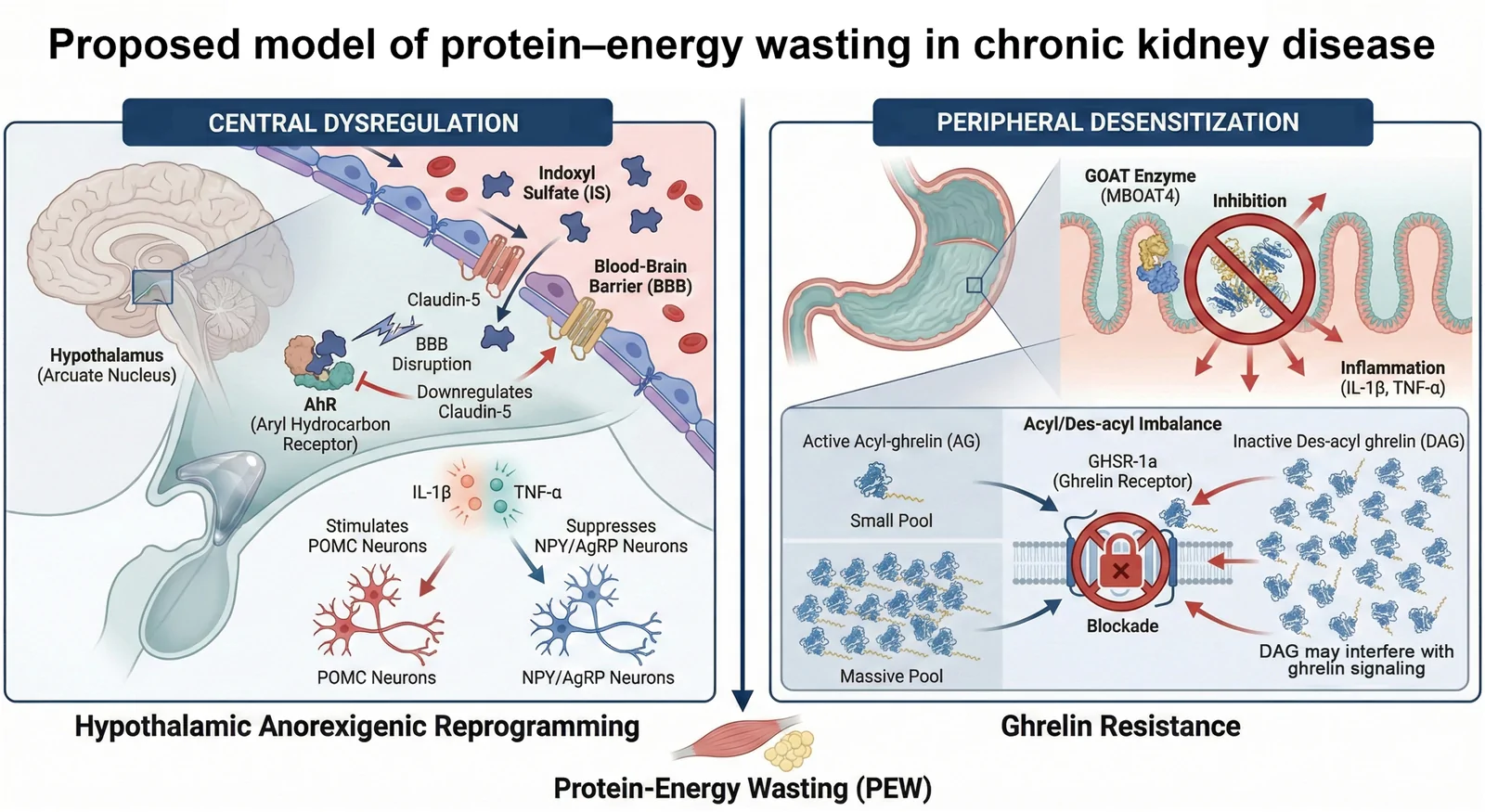
Proposed model of PEW in CKD, involving central dysregulation and peripheral desensitization of appetite signaling. Central dysregulation: Uremic toxins, such as IS, may contribute to BBB dysfunction, potentially through activation of the AhR pathway and alterations in tight junction proteins (e.g., claudin-5). BBB impairment may facilitate neuroinflammation, with increased cytokines (e.g., IL-1*β*, TNF-*α*) that are associated with altered hypothalamic signaling. These changes may shift the balance toward anorexigenic pathways, including increased activity of POMC neurons and reduced activity of NPY/AgRP neurons, thereby contributing to anorexia in CKD. Peripheral desensitization: In CKD, ghrelin signaling may be impaired due to alterations in acyl-ghrelin/DAG balance, reduced ghrelin activation by GOAT (MBOAT4), inflammation, and receptor-level resistance. Accumulation of DAG may interfere with ghrelin receptor signaling, contributing to functional ghrelin resistance. Together, these peripheral alterations may reduce orexigenic signaling and promote negative energy balance. The combined effects of central anorexigenic reprogramming and peripheral ghrelin resistance may contribute to the development of PEW in CKD. AgRP, agouti-related peptide; AhR, aryl hydrocarbon receptor; BBB, blood–brain barrier; CKD, chronic kidney disease; DAG, des-acyl ghrelin; GHSR-1a, growth hormone secretagogue receptor–1a; GOAT, ghrelin O-acyltransferase; IS, indoxyl sulfate; MBOAT4, membrane-bound O-acyltransferase domain containing 4; NPY, neuropeptide Y; PEW, protein-energy wasting; POMC, pro-opiomelanocortin.

**TABLE 1:** The neuroendocrine mechanisms in CKD

Mechanism	Physiological target	Pathological alteration in CKD	Metabolic consequence	Relevant references
Central dysregulation	Hypothalamus (arcuate nucleus)	Inflammation (IL-1*β*, TNF-*α*) suppresses NPY/AgRP and stimulates POMC/MC4-R	Anorexia, early satiety, and increased sympathetic drive (hypermetabolism)	[[18], [19], [20], [21]]
	BBB	Indoxyl sulfate activates endothelial AhR, downregulating claudin-5	Disruption of BBB integrity; influx of uremic toxins and cytokines into the CNS (“toxic brain”)	[22,23]
Peripheral desensitization	Ghrelin system	Suppression of GOAT enzyme by inflammation; accumulation of des-acyl ghrelin	“Ghrelin resistance”: Failure to convert pro-ghrelin to active acyl-ghrelin; blockade of hunger signals	[[24], [25], [26]]

Central dysregulation focuses on appetite suppression and neurotoxicity; peripheral desensitization focuses on the failure of peripheral utilization and hormonal signaling.

Abbreviations: AgRP, agouti-related peptide; AhR, aryl hydrocarbon receptor; BBB, blood–brain barrier; CKD, chronic kidney disease; CNS, central nervous system; GOAT, ghrelin O-acyltransferase; MC4-R, melanocortin-4 receptor; NPY, neuropeptide Y; POMC, pro-opiomelanocortin.

### Central dysregulation: the hypothalamic blockade

The hypothalamus serves as the command center for energy homeostasis, integrating peripheral signals of long-term energy status (leptin and insulin) and short-term hunger (ghrelin) to regulate feeding behavior via the arcuate nucleus [27]. In CKD, this delicate integration is disrupted by a combination of inflammation and neurotoxicity.

#### The inflammatory override

Chronic inflammation is a hallmark of CKD, driven by the retention of uremic toxins, oxidative stress, and gut dysbiosis-induced endotoxemia [[28], [29], [30]]. Proinflammatory cytokines, specifically IL-6, TNF-*α*, and IL-1*β*, are chronically elevated in the circulation of patients with CKD [31]. These cytokines act on the hypothalamus to fundamentally alter the “set point” of body weight [18]. Mechanistically, inflammation disrupts the balance between orexigenic [neuropeptide Y (NPY)/agouti-related peptide (AgRP)] and anorexigenic [pro-opiomelanocortin (POMC)/cocaine- and amphetamine-regulated transcript] neuropeptides in the arcuate nucleus [32].

In the uremic milieu, cytokines, such as IL-1*β*, bind to receptors on the vascular endothelium of the hypothalamus in rat models, triggering the release of local inflammatory mediators, such as tryptophan and serotonin [19,33]. This neuroinflammation suppresses the activity of NPY/AgRP neurons while stimulating the POMC pathway [20,34]. Crucially, aberrant signaling through the MC4-R—the downstream target of POMC—has been implicated as a potential contributor to cachexia. The activation of MC4-R not only suppresses appetite but also increases sympathetic nervous system outflow, driving the hypermetabolism and lipolysis observed in wasting [21,35]. This central resistance may attenuate the central response to energy deficit; even as muscle mass depletes, the hypothalamus perceives a state of “excess” or “toxicity” that necessitates fasting—a phenomenon often described as “sickness behavior”.

#### BBB integrity disruption by indoxyl sulfate

Experimental studies suggest that uremic toxins may alter BBB integrity, allowing penetration into the central nervous system (CNS).

The protein-bound uremic toxin indoxyl sulfate (IS) has been identified as a specific disruptor of BBB integrity. IS is an agonist of the aryl hydrocarbon receptor (AhR), a cytosolic ligand-activated transcription factor [14,22]. Upon binding IS, the AhR translocates to the nucleus of brain endothelial cells. This activation downregulates the expression of critical tight junction proteins, particularly claudin-5, and disrupts the actin cytoskeleton [23]. The loss of endothelial integrity increases the permeability of the BBB to other uremic solutes and inflammatory cytokines, exacerbating hypothalamic inflammation. Furthermore, AhR activation within the CNS has been linked to oxidative stress and cognitive impairment, contributing to the apathetic and lethargic phenotype often seen in advanced uremia. In in vitro endothelial cell cultures and rodent models of CKD, blocking AhR or using AhR-knockout mice prevents IS-induced BBB disruption and cognitive decline, directly linking this toxin-receptor interaction to central dysfunction, although human validation remains pending [23,36].

### Peripheral desensitization: the ghrelin axis failure

Ghrelin, the only known circulating orexigenic hormone [37], is typically elevated in fasting states to trigger feeding. In CKD, total ghrelin levels are often paradoxically high, yet patients remain anorectic. This suggests a state of “ghrelin resistance” analogous to insulin resistance in type 2 diabetes [24,25].

#### The acyl/des-acyl ratio imbalance

Acyl-ghrelin (AG) is strictly required for binding the growth hormone secretagogue receptor–1a (GHSR-1a) to stimulate appetite [38,39]. Des-acyl ghrelin (DAG) is the unacylated form, which accounts for 80%–90% of circulating ghrelin. It does not bind GHSR-1a and was historically considered inert. However, recent evidence suggests that DAG may actively antagonize the metabolic effects of AG and can cross the BBB to exert independent effects, potentially unrelated to appetite stimulation [26,40].

In CKD, the ratio of AG to DAG may be altered. Although total ghrelin elevation may occur across advanced CKD, alterations in the AG/DAG balance have been associated with nutritional or wasting phenotypes in some studies [[41], [42], [43], [44]]. High levels of DAG have been shown to correlate with lower body fat mass and insulin resistance in other metabolic conditions [[41], [42], [43]], and in the uremic context, the altered DAG signaling may interfere with ghrelin signaling across the BBB or its binding at central receptors and may attenuate orexigenic signaling.

#### The GOAT enzyme defect

The acylation of ghrelin is catalyzed by the enzyme GOAT, also known as membrane-bound O-acyltransferase domain containing 4. This enzyme is the rate-limiting step in the production of active ghrelin [45]. GOAT requires available medium-chain fatty acids (specifically octanoate) to acylate pro-ghrelin [46]. In uremia, dietary intake of lipids may be reduced, or metabolic alterations may limit the availability of this specific substrate. More critically, systemic inflammation has been shown to rapidly downregulate the ghrelin-GOAT system. In models of lipopolysaccharide-induced inflammation (mimicking the endotoxemia of CKD), plasma AG and GOAT protein levels drop significantly within hours, preceding the onset of fever and anorexia [26]. This suggests that the “shutting off” of ghrelin activation via GOAT suppression is a coordinated component of the acute phase response [47]. Although direct inhibition of GOAT by uremic toxins like IS is an area of active investigation, the correlation between inflammation and reduced AG/DAG ratios supports the hypothesis that the uremic milieu functionally impairs this enzyme [24]. The physiological significance of the elevated total ghrelin pool in CKD remains uncertain.

## Mediators of Metabolic Disruption: Toxins and Cytokines

Specific molecular mediators, accumulating due to renal failure, shift metabolism from anabolism to catabolism (Table 2; Figure 2).

**TABLE 2:** Key molecular mediators and their metabolic targets

Mediator	Origin	Primary target receptor/pathway	Effect on muscle/adipose	Relevant references
Indoxyl sulfate	Gut microbiome (tryptophan metabolism)	Aryl hydrocarbon receptor	Upregulates myostatin and atrogin-1; induces mitochondrial ROS; disrupts BBB	[[48], [49], [50]]
GDF15	Stress response (kidney/liver/muscle)	GFRAL–RET (brainstem)	Induces significant anorexia ("sickness behavior"); Drives lipolysis in adipose tissue	[[51], [52], [53], [54]]
Myostatin/activin A	Muscle/systemic	ActRIIB (activin receptor)	Phosphorylates Smad2/3; blocks Akt/mechanistic target of rapamycin (mTOR) (protein synthesis); activates UPS (degradation)	[[55], [56], [57]]
Angiotensin II	RAAS activation	AT1 receptor	Induces UPS via caspase-3 activation; suppresses IGF-1 signaling; increases REE	[58]

Indoxyl sulfate levels are effectively cleared by healthy kidneys but reach toxic levels in ESRD due to failed tubular secretion. GDF15 acts as a stress-responsive cytokine of the TGF-*β* superfamily.

Abbreviations: ActRIIB, activin receptor type IIB; BBB, blood–brain barrier; ESRD, end-stage renal disease; GDF15, growth differentiation factor 15; GFRAL, GDNF family receptor *α*-like; IGF-1, insulin-like growth factor–1; RAAS, renin-angiotensin-aldosterone system; REE, resting energy expenditure; RET, rearranged during transfection; ROS, reactive oxygen species; Smad, small mothers against decapentaplegic; TGF-*β*, transforming growth factor-*β*; UPS, ubiquitin–proteasome system.

**FIGURE 2:**
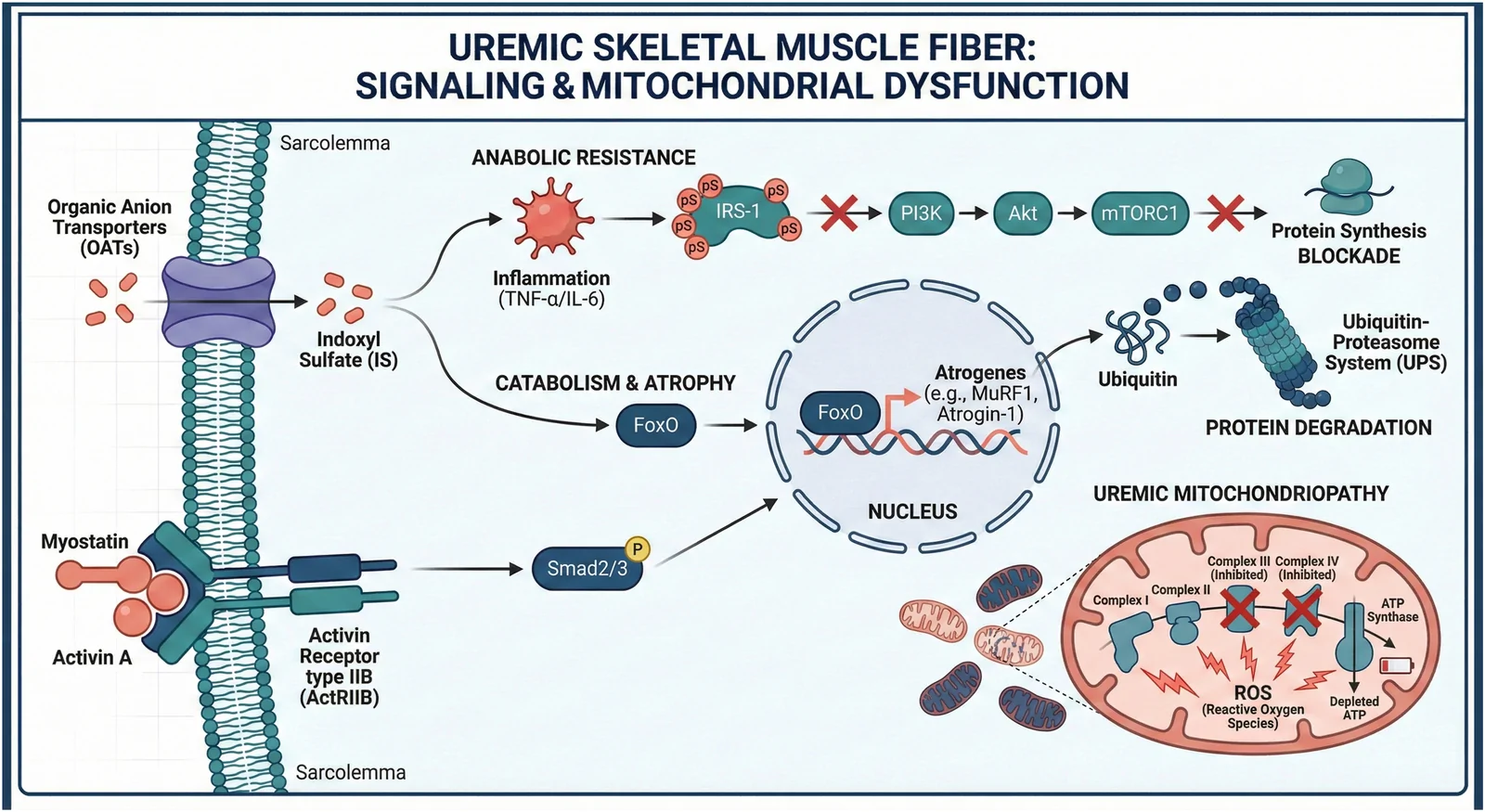
The proposed mechanisms of anabolic resistance, protein degradation, and mitochondrial dysfunction in uremic skeletal muscle. Uremic toxins, including IS, may enter skeletal muscle cells via OATs or related transport systems and contribute to inflammation and metabolic stress. Inflammatory signaling (e.g., TNF-*α*, IL-6) is associated with impaired insulin/IGF-1 signaling, resulting in reduced IRS-1/PI3K/Akt/mTORC1 activity and decreased protein synthesis, a condition referred to as anabolic resistance. In parallel, catabolic pathways may be activated through FoxO transcription factors, leading to increased expression of aerogenes (e.g., MuRF1, atrogin-1) and activation of the ubiquitin–proteasome system, thereby promoting protein degradation. Myostatin/activin signaling via ActRIIB–Smad2/3 may further enhance muscle atrophy-related gene expression. Mitochondrial dysfunction is commonly observed in CKD and may involve impaired electron transport chain activity, increased ROS generation, and reduced ATP production. These alterations may contribute to muscle weakness, metabolic inefficiency, and progression of protein-energy wasting. ActRIIB, activin receptor type IIB; CKD, chronic kidney disease; FoxO, forkhead box O; IGF-1, insulin-like growth factor–1; IRS-1, insulin receptor substrate–1; IS, indoxyl sulfate; mTORC1, mechanistic target of rapamycin complex 1; OAT, organic anion transporter; PI3K, phosphoinositide 3-kinase; ROS, reactive oxygen species; Smad, small mothers against decapentaplegic; UPS, ubiquitin–proteasome system; MuRF1, muscle RING-finger protein-1.

### Uremic toxins: the IS connection

Among the diverse array of uremic retention solutes, IS stands out as a premier metabolic disruptor. IS accumulates significantly from normal levels (<2.5 μM) to uremic levels (>100–200 μM) in advanced ESRD. Concentrations in this range have been used in experimental studies investigating AhR activation and cellular effects, although the relevance of these experimental concentrations to tissue exposure in humans remains uncertain [48,59,60]. IS penetrates skeletal muscle cells via organic anion transporters [61,62]. Once intracellular, it exerts toxicity through multiple pathways:

AhR-genomic pathway: As noted in the brain, IS binds to the cytosolic AhR in murine and human skeletal muscle cells [61,63]. The IS-AhR complex translocates to the nucleus and dimerizes with the AhR nuclear translocator. This complex binds to dioxin response elements in the promoter regions of target genes [49,64]. Crucially, IS-AhR activation has been shown to upregulate the expression of myostatin (a potent inhibitor of muscle growth) [50].

Oxidative stress: IS induces the production of superoxide and other reactive oxygen species (ROS) within the myocyte [65]. This oxidative burst activates the nuclear factor kappa B pathway, which further drives the expression of inflammatory cytokines (IL-6, TNF-*α*) locally within the muscle, creating a vicious autocrine loop of inflammation and catabolism [66,67]. Interestingly, AhR activation by IS also upregulates P-glycoprotein/ATP-binding cassette subfamily B member 1 expression in the liver and potentially other tissues [68]. This may have implications for the pharmacokinetics of drugs used in CKD, potentially increasing the clearance of substrates (like cyclosporine) or altering the distribution of therapeutic agents intended to treat cachexia.

### GDF15: the stress hormone and “sickness” driver

GDF15 has rapidly emerged as a critical biomarker and driver of cachexia in chronic diseases, including CKD and cancer. GDF15 is a stress-responsive cytokine of the transforming growth factor-*β* (TGF-*β*) superfamily [13,51]. Its levels rise dramatically in response to mitochondrial dysfunction, cellular injury, and uremia [52,69].

GDF15 acts through the specific GFRAL receptor and its coreceptor, rearranged during transfection (receptor tyrosine kinase) (RET), which are expressed almost exclusively in the area postrema of the brainstem and nucleus of the solitary tract [53]. The area postrema is a circumventricular organ with a permeable BBB, making it accessible to circulating GDF15 [70]. Activation of the GFRAL–RET complex triggers an aversive response that suppresses appetite and induces nausea. This is likely an evolutionary adaptation to prevent the ingestion of toxins, but in CKD, the endogenous “toxin” (uremia) triggers a chronic, maladaptive anorexia [51]. Beyond anorexia, GDF15 activates the sympathetic nervous system, increasing lipolysis and potentially driving the browning of white adipose tissue (WAT), which increases futile energy expenditure [54]. Although elevated GDF15 has been reported across CKD populations, higher concentrations have been associated with measures of wasting and adverse clinical outcomes in some cohorts [51,71].

### The activin/myostatin axis

In CKD, the balance between myogenic factors and negative regulators like myostatin and activin A is lost.

Metabolic acidosis, glucocorticoids, and angiotensin II—all common in CKD—synergistically upregulate the expression of myostatin and activin A in rodent and human skeletal muscle tissue [72,73]. Furthermore, angiotensin II acts via the AT1 receptor to suppress anabolic IGF-1 signaling, triggers the ubiquitin–proteasome system (UPS) through caspase-3 activation, and drives hypermetabolism by increasing resting energy expenditure [58]. These ligands bind to the activin receptor type IIB (ActRIIB) on the myocyte membrane. This recruits ALK4/5 type I receptors and leads to the phosphorylation of small mothers against decapentaplegic (Smad) 2 and Smad3 transcription factors [55]. Phosphorylated Smad2/3 translocates to the nucleus, where it suppresses the expression of myogenic regulatory factors (myoblast determination protein 1, myogenin) and inhibits the Akt/mTOR pathway. This blockade effectively prevents protein synthesis [71].

## Metabolic Consequences: The Cellular Engine Failure

The convergence of central anorexia and peripheral signaling toxins results in deep metabolic derangements within skeletal muscle and adipose tissue. The clinical manifestation is not just “small muscle” but “sick muscle”—tissue that is bioenergetically compromised and resistant to repair.

### Anabolic resistance: the phosphoinositide 3-kinase/Akt/mTOR impairment

In CKD, the anabolic response to protein or insulin via the phosphoinositide 3-kinase (PI3K)/Akt/mechanistic target of rapamycin complex 1 (mTORC1) cascade is blunted, termed “anabolic resistance” [74,75].

Insulin signaling defect: Uremic toxins and inflammatory cytokines (TNF-*α*) induce serine phosphorylation of IRS-1 in skeletal muscle. This pathological phosphorylation prevents IRS-1 from associating with PI3K, effectively uncoupling the insulin receptor from its downstream effectors [76,77]. Consequently, the activation of Akt (protein kinase B) is impaired. Akt is the important upstream regulator that activates mTORC1 [78]. Without robust Akt signaling, mTORC1 fails to phosphorylate its targets, p70S6K and 4E-BP1, which are necessary for initiating mRNA translation and protein synthesis [79,80]. Akt also has a critical role in suppressing catabolism by phosphorylating forkhead box O (FoxO) transcription factors, which exports them from the nucleus. In the absence of Akt activity, FoxO remains nuclear, driving the significant upregulation of the UPS. This creates a “double-negative”: protein synthesis is turned off, and protein degradation is turned on [81,82].

### Mitochondrial dysfunction: the bioenergetic crisis

A specific “uremic mitochondriopathy” may impair oxidative phosphorylation and precede gross atrophy [83].

Experimental studies suggest that uremic metabolites, including IS, indole-3-acetic acid, and kynurenine, have been shown to directly inhibit the activity of the electron transport chain (ETC), specifically complexes III and IV. This “chemical hypoxia” reduces the capacity for ATP generation, leading to the substantial fatigue and reduced exercise tolerance seen in patients with CKD [16,84]. The blockade of the ETC causes electron leakage, generating superoxide anions. This ROS damages mitochondrial DNA and membrane lipids (cardiolipin), further impairing function [85,86]. Mitochondrial health depends on a balance between fusion (joining mitochondria to share resources) and fission (splitting to isolate damage). In CKD, the balance tips may shift toward fission. There is an upregulation of fission proteins (dynamin-related protein 1, mitochondrial fission 1 protein) and a downregulation of fusion proteins (mitofusin-2, optic atrophy 1 protein) [87,88]. This results in a fragmented network of small, inefficient mitochondria. Normally, damaged mitochondria are cleared by mitophagy (a selective form of autophagy). In CKD, this process is often impaired or overwhelmed by the sheer volume of damage, leading to the accumulation of dysfunctional mitochondria that release pro-apoptotic factors (cytochrome c) and trigger muscle fiber death [89].

Crucially, impaired mitochondrial bioenergetics may directly contribute to PEW. Specifically, reduced ATP-generating capacity may constrain energy-demanding anabolic processes, whereas excess mitochondrial oxidative stress may promote FoxO/UPS–associated muscle proteolysis. It is important to emphasize the experimental nature of much of this mechanistic evidence, as these specific pathways have not yet been definitively established in humans with CKD.

### Adipose tissue: the lipolytic drive

As demonstrated in human adipocytes, WAT undergoes “browning”—a phenotypic switch to a beige/brown thermogenic phenotype [90]—driven by uremic toxins and elevated parathyroid hormone [91,92]. This increases futile energy expenditure. Furthermore, the suppression of insulin signaling in adipocytes unleashes hormone-sensitive lipase, driving lipolysis and the release of free fatty acids [93]. Although this loss of fat mass might seem distinct from sarcopenia, the loss of adiposity removes a critical energy reserve and is strongly associated with mortality in the ESRD population (the “obesity paradox”) [94].

## Comparative Pathophysiology: Distinct Metabolic Architectures of Wasting

Although the “wasting” phenotype appears uniform across chronic diseases, underlying metabolic architectures differ critically, necessitating disease-specific strategies (Table 3).

**TABLE 3:** Comparative metabolic architecture of wasting syndromes

Feature	CKD (PEW)	Cancer cachexia	Heart failure cachexia	COPD cachexia	Relevant references
Primary driver	Uremic toxins (indoxyl sulfate, p-cresol), metabolic acidosis	Tumor factors (PIF, LMF), systemic inflammation	Hemodynamic (gut edema, venous congestion)	Hypoxia, oxidative stress, work of breathing	[48,[95], [96], [97]]
Blood–brain barrier	Potentially disrupted; supported primarily by experimental evidence	Generally intact	Intact	Intact	[22]
Resting energy expenditure	Variable/hypometabolic (due to inactivity)	Hypermetabolic (tumor demand, futile cycling)	Hypermetabolic (cardiac workload)	Hypermetabolic (respiratory mechanics)	[[98], [99], [100]]
Fluid status	Volume overload (masks wasting)	Euvolemic/dehydrated	Volume overload	Euvolemic	[101]
Reversibility	Unknown; proposed to depend on correction of metabolic barriers	Refractory (unless tumor removed)	Low	Moderate	[102,103]
GDF15 response	Elevated in CKD; association with PEW/wasting requires further phenotypic validation	High levels; responds to inhibition	Elevated; inhibition harmful (GARDEN-TIMI 74)	Elevated	[51]

PEW in CKD is often “occult” because volume overload can mask the physical loss of muscle mass. GARDEN-TIMI 74 refers to the 2025 trial investigating GDF15 inhibition in heart failure (NCT05492500).

Abbreviations: CKD, chronic kidney disease; COPD, chronic obstructive pulmonary disease; GDF15, growth differentiation factor 15; LMF, lipid mobilizing factor; PEW, protein-energy wasting; PIF, proteolysis-inducing factor.

Unlike cancer cachexia driven by tumor-host crosstalk [95,104,105], CKD PEW may be driven by accumulated uremic toxins (e.g., IS) and potential BBB disruption. Furthermore, unlike hypermetabolic states in cancer or chronic obstructive pulmonary disease (COPD) [96,106], the CKD profile is frequently normometabolic or hypometabolic because of inactivity [98].

This clinical impasse is further confounded by occult wasting, where volume overload can mask significant muscle loss—a feature shared with heart failure but distinct from the visible weight loss in cancer and COPD [101]. Consequently, standard nutritional interventions or therapies directly extrapolated from oncology (e.g., GDF15 antagonists) [51] carry distinct risks in the cardiorenal uremic milieu and demand disease-specific validation.

## Implications for Nutritional Therapy: From Mechanism to Intervention

The elucidation of these mechanisms suggests the need for a potential shift in our therapeutic approach. Currently, the standard of care for PEW in CKD follows the Kidney disease outcomes quality initiative (KDOQI) 2020 clinical practice guidelines [5]. These guidelines emphasize optimization of dietary intake through counseling, ONS, and IDPN. Recommendations focus on ensuring adequate protein intake (1.2 g/kg body weight/d for dialysis patients) and energy provision (30–35 kcal/kg/d) to maintain nitrogen balance. Although these interventions can provide necessary substrate, they primarily address the “supply” side of the equation.

Standard nutritional therapy often fails to reverse wasting [107] because it does not dismantle the hormonal and metabolic barriers described above. The patient receives fuel (calories/protein), but the engine (muscle synthesis) remains blocked by anabolic resistance and inflammation.

### Exercise as a metabolic sensitizer

Before administering specific nutritional or pharmacological agents, physical activity—particularly resistance training—may serve as a foundational “sensitizer.” Exercise mechanically stimulates the Akt/mTORC1 pathway in mammals [108,109], potentially bypassing uremic anabolic resistance. Furthermore, it may modulate systemic inflammation and improve insulin sensitivity [110,111], thereby priming the skeletal muscle to effectively utilize subsequent nutritional supplementation.

### Nutrient-hormone crosstalk: reprogramming the uremic environment

Leveraging specific nutrients may help reprogram the disrupted neuroendocrine axes of CKD.

Omega-3 (*n*–3) fatty acids and barrier integrity: The chronic neuroinflammation driven by cytokines like IL-1*β* suppresses NPY/AgRP hunger neurons and stimulates the anorexigenic POMC pathway [112]. *ω*–3 fatty acids, particularly EPA and DHA, have been investigated for their potential to modulate inflammation and anabolic signaling. By attenuating hypothalamic neuroinflammation, these fatty acids have the potential to stabilize the BBB, which is frequently compromised in CKD by IS-mediated AhR activation [113,114]. Stabilizing this barrier reduces the influx of uremic toxins and cytokines into the CNS, potentially restoring appetite signaling by making the brain less “deaf” to the body's energy deficits.

Branched-chain amino acids (BCAAs) and anabolic resistance: Uremic muscle wasting is characterized by a “leucine trigger” deficit, where skeletal muscle becomes refractory to anabolic stimuli. This anabolic resistance is driven by uremic toxins that induce serine phosphorylation of IRS-1, effectively uncoupling the insulin receptor from its downstream PI3K/Akt/mTORC1 signaling cascade [115]. Supplementation with BCAAs or their metabolites, such as *β*-hydroxy *β*-methylbutyrate, is designed to bypass this blockade [116]. These nutrients aim to directly stimulate protein synthesis and overcome the “anabolic resistance” that causes the uremic body to metabolize its own skeletal muscle even when exogenous nutrients are available.

Integration with hormonal resistances: These nutritional interventions are not merely caloric but are designed to dismantle the specific hormonal blockades. *ω*–3s target the central “hypothalamic blockade” by reducing the inflammatory override of the appetite set point. Meanwhile, BCAAs target the peripheral “anabolic resistance,” helping to release the metabolic brake on muscle growth even in the presence of silencing signals from the insulin and ghrelin axes. Only by reconnecting nutrient intake with these homeostatic signals can nutritional therapy bridge the survival gap for patients with advanced CKD.

### Pharmacological interventions: lessons from cancer cachexia

Emerging pharmacological interventions developed for cancer cachexia require CKD-specific trials before extrapolation (Table 4):

**TABLE 4:** Emerging pharmacological interventions and safety profiles

Agent	Class/mechanism	Status in CKD	Key risk in renal population	Relevant references
Anamorelin	Ghrelin agonist (GHSR-1a)	Investigational (approved in Japan for cancer)	Hyperglycemia: high risk in diabetic nephropathy; accumulation of drug metabolites	[117,118]
Ponsegromab	GDF15 antagonist (anti-GDF15 mAb)	Preclinical/phase 2; 3 (cancer)	Cardiac safety: worsening HF seen in GARDEN-TIMI 74 trial; caution in cardiorenal syndrome	[51,119,120]
Bimagrumab	ActRII antagonist (myostatin/Activin blocker)	Phase 2 (obesity/diabetes)	Muscle cramps/diarrhea: potential for fluid/electrolyte imbalance in dialysis patients	[121,122]
TCMCB07	MC4-R antagonist	Preclinical (animal models)	Sympathetic tone: must verify lack of pressor effects (HTN) in CKD	[123,124]

Anamorelin safety is of particular concern in the CKD population because of the high prevalence of diabetic nephropathy. Bimagrumab is being investigated for its unique ability to reduce fat mass while increasing lean mass.

Abbreviations: ActRII, activin receptor type II; CKD, chronic kidney disease; GDF15, growth differentiation factor 15; GHSR-1a, growth hormone secretagogue receptor–1a; HTN, hypertension; mAb, monoclonal antibody; MC4-R, melanocortin-4 receptor.

#### Ghrelin receptor agonists (anamorelin)

Anamorelin is an oral selective agonist of the GHSR-1a receptor, designed to mimic AG and stimulate appetite and growth hormone release [117]. Although oncology trials have demonstrated its ability to significantly increase lean body mass (LBM) and body weight [[125], [126], [127]], in CKD, it could potentially bypass the “ghrelin resistance” caused by DAG. An important safety consideration is hyperglycemia. Recent reports indicate that anamorelin can induce or worsen diabetes [118], a finding particularly risky for the CKD population where diabetic nephropathy is a leading cause of ESRD. Furthermore, trials have consistently shown a dissociation between mass gain and functional improvement (handgrip strength), suggesting the “quality” of muscle gained may remain poor.

#### GDF15 antagonists (ponsegromab)

Ponsegromab is a monoclonal antibody that neutralizes GDF15, preventing it from binding to the GFRAL receptor in the brainstem [119]. Having shown efficacy in reversing weight loss in phase 2 cancer trials, [120] neutralizing this “sickness signal” could potentially improve appetite-related outcomes in selected patients with ESRD, where GDF15 levels are highly elevated, but this hypothesis requires direct evaluation. Phase 3 trials are planned, but specific safety data in patients with reduced renal clearance are currently lacking.

#### MC4-R antagonists (TCMCB07)

These peptides block the anorexigenic signaling driven by inflammation in the hypothalamus [123]. In rat models of CKD and cancer cachexia, TCMCB07 administration preserved lean mass, reduced hypothalamic inflammation, and restored food intake [124]. By dampening the central sympathetic drive, it may also reduce hypermetabolism.

#### Myostatin/activin inhibition

Targeting the TGF-*β* superfamily has yielded mixed results, highlighting the complexity of this pathway.

Bimagrumab: This monoclonal antibody blocks the ActRII, preventing the binding of myostatin, activin A, and growth differentiation factor 11 (GDF11) [121]. In trials for obesity and sarcopenia, bimagrumab has shown potent effects in reducing fat mass and increasing lean mass, a profile particularly attractive for “sarcopenic obesity” [121,128]. However, functional improvements in older adults have been inconsistent [122,129].

In contrast, PINTA-745, a “peptibody” designed to neutralize myostatin alone, failed to meet its primary endpoints for LBM accretion in a phase 2 trial of patients with ESRD [130]. The failure of PINTA-745 suggests that inhibition of myostatin alone may be insufficient in ESRD, although differences in study populations and mechanisms of action should be considered. The ActRII receptor integrates signals from multiple ligands (activin A, GDF11), and blocking the receptor itself (or using a ligand trap) may be necessary to achieve a clinically meaningful blockade [131].

In summary, although the pipeline of agents is promising, the transition from cancer cachexia to CKD management warrants significant caution. The “sensitize-then-feed” paradigm—using these agents to lower the barrier before aggressive feeding—remains the testable conceptual framework for future trials.

In conclusion, the “nutritional paradox” in CKD may reflect broader disturbances in metabolic and homeostatic signaling. The body in uremia is not simply hungry; it is characterized by substantial dysregulation of hormonal and metabolic signaling.

Current research has mapped this blockade to a mechanism: Centrally, uremic toxins like IS breach the BBB, whereas cytokines and GDF15 reprogram the hypothalamus to suppress appetite. Peripherally, anabolic signaling through the Akt/mTOR pathway may be impaired by insulin resistance and ActRII activation, whereas its catabolic machinery (UPS/atrogenes) is hyperactivated. Simultaneously, mitochondrial function may be impaired by uremic solutes, leading to bioenergetic failure.

This understanding clarifies why single-agent interventions or simple caloric supplementation have historically failed. The path forward lies in multimodal precision medicine, shifting the clinical focus from “dietary prescription” to “metabolic rehabilitation.” A coordinated “sensitize-then-feed” framework—using exercise, *ω*–3s, and targeted agents to lower metabolic barriers before aggressive feeding—provides a testable conceptual framework for future studies. Although agents, such as ponsegromab, anamorelin, or ActRII antagonists, offer a promising pipeline for restoring appetite and releasing the “brake” on muscle growth, their integration into CKD management warrants extreme caution. Specific trials are needed to ensure that these interventions do not exacerbate the unique cardiorenal risks or glycemic instabilities inherent to the uremic milieu. Further investigation is needed to determine whether modifying these pathways can improve the response to nutritional therapy and clinically relevant outcomes.

We must explicitly acknowledge the limitations of the current knowledge base. Much of the mechanistic framework proposed herein, particularly regarding BBB disruption and specific organelle-level events, relies heavily on preclinical in vitro and rodent models. Definitive validation of these pathways in human CKD cohorts remains pending.

To bridge this translational gap and test the proposed “sensitize-then-feed” hypothesis, specific future studies are warranted. We propose the initiation of phase II proof-of-concept trials combining IDPN with targeted metabolic modulators (such as GDF15 neutralizing antibodies) in strictly defined PEW cohorts. Furthermore, translational clinical studies utilizing advanced neuroimaging, such as dynamic contrast-enhanced MRI, could provide a means to evaluate BBB permeability and central neuroinflammation in human patients with CKD.

## Author contributions

The authors’ responsibilities were as follows – BY, CX: contributed to conceptualization and validation; BY, ML: contributed to writing – original draft; ZC, N Lan, FZ, N Liu: contributed to writing – review and editing; and all authors: read and approved the final version of the manuscript.

## Data availability

Data sharing is not applicable to this article as no new data were created or analyzed in this study.

## Funding

The authors reported no funding received for this study.

## Conflict of interest

The authors report no conflicts of interest.
